# Trehalose versus carboxymethylcellulose oral spray for relieving radiation-induced xerostomia in head and neck cancer patients: a randomized controlled trial

**DOI:** 10.1186/s12903-023-02966-4

**Published:** 2023-05-13

**Authors:** Pornpan Piboonratanakit, Joao N. Ferreira, Kulpriya Pravinvongvuthi, Khwanchanok Maison, Ganokon Urkasemsin, Thirayu Boonroung, Anussara Prayongrat, Chawalit Lertbutsayanukul, Jeerus Sucharitakul, Anjalee Vacharaksa

**Affiliations:** 1 Department of Oral Medicine, Faculty of Dentistry, Chulalongkorn University, Bangkok, Thailand; 2 Research Unit in Oral Diseases, Faculty of Dentistry, Chulalongkorn University, Bangkok, Thailand; 3 Avatar Biotechnologies for Oral Health and Healthy Longevity Research Unit, Department of Research Affairs, Faculty of Dentistry, Chulalongkorn University, Bangkok, Thailand; 4 Geriatric and Special Patients (International) Program, Faculty of Dentistry, Chulalongkorn University, 34 Henri-Dunant Road, Pathumwan, Bangkok, 10330 Thailand; 5 Department of Preclinic and Applied Animal Science, Faculty of Veterinary Science, Mahidol University, Bangkok, Thailand; 6 Dental Department, King Chulalongkorn Memorial Hospital, Bangkok, Thailand; 7 Division of Radiation Oncology, Department of Radiology, Faculty of Medicine, Chulalongkorn University, Bangkok, Thailand; 8 Department of Biochemistry, Faculty of Dentistry, Chulalongkorn University, Bangkok, Thailand; 9 Department of Microbiology, Faculty of Dentistry, Chulalongkorn University, Bangkok, Thailand; 10 Excellent Center on Microbiology and Immunology, Faculty of Dentistry, Chulalongkorn University, Bangkok, Thailand

**Keywords:** Head and neck cancer, Radiotherapy, Salivary gland, Hyposalivation, Xerostomia, Trehalose

## Abstract

**Background:**

The aim of this study was to investigate the effect of trehalose oral spray to relieve radiation-induced xerostomia on a randomized controlled trial (RCT).

**Methods:**

Prior to RCT, the effect of trehalose (5–20%) on the epithelial growth of fetal mouse salivary gland (SG) explants was evaluated to confirm if 10% trehalose exerted the best epithelial outcomes. Participants who completed radiotherapy for head and neck cancer (HNC) treatment were enrolled in a double-blind RCT, according to inclusion and exclusion criteria as per the CONSORT statement. The experimental group (n = 35) received 10% trehalose spray, while the control group (n = 35) received carboxymethylcellulose (CMC) spray to apply intra-orally 4 times/day for 14 days. Salivary pH and unstimulated salivary flow rate were recorded pre- and post-interventions. The Xerostomia-related Quality of Life scale (XeQoLs) was filled, and scores assessed post-interventions.

**Results:**

In the SG explant model, pro-acinar epithelial growth and mitosis was supported by 10% topical trehalose. As for RCT outcomes, salivary pH and unstimulated salivary flow rate were significantly improved after use of 10% trehalose spray when compared to CMC (*p* < 0.05). Participants reported an improvement of XeQoLs dimension scores after using trehalose or CMC oral sprays in terms of physical, pain/discomfort, and psychological dimensions (*p* < 0.05), but not social *(p* > 0.05). When comparing between CMC and trehalose sprays, XeQoLs total scores were not statistically different (*p* > 0.05).

**Conclusions:**

The 10% trehalose spray improved salivary pH, unstimulated salivary flow rate, and the quality-of-life dimensions linked with physical, pain/discomfort, and psychological signs. The clinical efficacy of 10% trehalose spray was equivalent with CMC-based saliva substitutes for relieving radiation-induced xerostomia; therefore, trehalose may be suggested in alternative to CMC-based oral spray.**(**Thai Clinical Trials Registry; https://www.thaiclinicaltrials.org/ TCTR20190817004).

**Supplementary Information:**

The online version contains supplementary material available at 10.1186/s12903-023-02966-4.

## Introduction

Almost a million people are affected by head and neck cancers (HNC) worldwide [1]. Radiotherapy is a standard treatment for HNC; however, radiation also induces collateral damage to surrounding normal tissues and causes several complications including sore throat, altered taste, limited saliva flow, dental caries, as well as impaired speaking, chewing or swallowing functions. Salivary glands (SG) are often affected by radiation exposure causing a reduction in salivation and pH of secreted saliva [2, 3]. The total dose of radiotherapy for HNC ranges from 50 to 70 Gy, while doses over 52 Gy begin to impair SG function [4–6]. Thus, SG hypofunction is frequently reported in HNC patients treated with radiotherapy [6, 7]. Many reports showed that salivary flow rate decreases steeply after radiotherapy, and it may take 3 to 6 months until SG gradually recovers [7, 8]. Saliva plays a significant role as an oral lubricant to maintain oral health, facilitate masticatory functions, speaking and other normal daily activities [9]. Therefore, radiation-related SG hypofunction affects physiologic functions, nutritional intake causing weight loss, and compromised general health and quality of life.

Xerostomia is subjective perception of dry mouth associated with SG hypofunction [10]. Treatment approach for radiation-related xerostomia focuses on relieving symptoms including saliva substitutes or stimulants. However, saliva stimulants tend to have adverse effects such as sweating, dizziness, flushing of the face and neck, chills, or increased urge to urinate [11], whereas the saliva substitutes, for example in the form of oral rinse or moisturizing gel [12–14], were simple and effective to manage dry mouth [15, 16]. Saliva substitutes have been reported to physically coat oral tissues for moisture retention [11, 12]. Contents of saliva substitutes commonly are carboxymethylcellulose (CMC), mucins, xanthan gum, hydroxymethylcellulose, linseed oil, or polyethylene oxide. Most saliva substitutes contain CMC because CMC has high viscosity and mucoadhesive property that can retain coating on oral mucosa to improve dry mouth [16]. However, xylitol or sorbitol may be added to CMC-based saliva substitutes as a sweetener to improve its taste and increase patient compliance [13].

There are other natural moistening agents, such as trehalose, to keep the lining mucosa moisturized [17, 18]. Trehalose is a nonreducing disaccharide with two glucose units (1,1-glycosidic linkage). This sugar is reportedly synthesized by many organisms, including bacteria, yeast, fungi, insects, invertebrates, and lower and higher plants [18]. Trehalose enhances the desiccation endurance on mammalian cells [19, 20], and it is also a therapeutic agent for dehydration and oxidative stress [21, 22]. Trehalose has been approved for a variety of medical applications in ophthalmology [23]. Regarding its moistening properties, a previous study has shown that trehalose can protect corneal epithelial cells from dehydration as well as cells and their protein content from oxygen radical damage [20]. An adjuvant treatment of 3% trehalose was reported to prevent dry eye after Laser In-Situ Keratomileusis [24]. Although trehalose has been chemically defined as a sugar, it is not a substrate for glucosyltransferase and can inhibit the synthesis of water-insoluble glucan, all of which are relevant to control dental caries [25]. Taken together, trehalose is a non-cariogenic substance with moisture retention capabilities and sugar content. Trehalose may serve as a good alternative for saliva substitutes without any additional flavor enhancer.

The utilization of a moisturizing oral spray containing 10% trehalose solution has been reported for the first time in a preliminary study by Mori et al. [17] with only ten healthy patients. In the study by Mori et al., the recovery of oral moisture by trehalose solution was shown by the reduction of fungiform papillae shrinkage [17]. Consistent with these observations, Ota et al. [12] found dry mouth symptoms had remarkably improved after using micro-gel spray for one week. Despite these positive clinical outcomes on dry mucosal surfaces from previous preliminary studies, a limited understanding of such phenomena persists, and research questions remain as to what biological mechanisms and effects can trehalose exert on the SG and mucosal tissues. Both CMC- and trehalose-based oral spray appear to have moisture retention capability; however, a clinical comparison between trehalose and conventional CMC-based saliva substitutes has never been performed with radiation-induced xerostomia patients. The objective of this study was to compare the xerostomia-related treatment outcomes of 10% trehalose to be equivalent with the conventional CMC-based saliva substitutes for relieving radiation-induced xerostomia in a double-blind, randomized-controlled trial (RCT). The xerostomia-related quality of life scores reflecting physical, pain/discomfort, psychological and social dimension were collected for the primary outcome. Salivary pH and volume were collected as secondary outcomes.

## Materials and methods

### Clinical study ethics and sampled population

The study was approved by the Institutional Review Board of the Faculty of Medicine, Chulalongkorn University (IRB No.534/62), and registered in an international clinical trial register (Thai Clinical Trials Registry; https://www.thaiclinicaltrials.org/; TCTR20190817004; First submitted date 17/08/2019) prior to participant recruitment. From January to September 2020, patients from the Head and Neck Cancer Unit (at King Chulalongkorn Memorial Hospital) fulfilling specific inclusion and exclusion criteria were recruited with informed consent. Criteria for inclusion were patients over 18 years of age who previously completed radiotherapy course of 51–70 Gy with the fields of radiation encompassing the major and minor salivary glands for at least 1 month. Exclusion criteria were patients with Sjögren’s syndrome or other salivary gland diseases, being uncooperative, nor on feeding tube. The number of participants was calculated using G*Power software based on the study of Gerardo Gormez-Moreno et al. [26], at the significant level (α) of 0.05, and the power (1-β) of 0.8. A drop-out rate was estimated at 10%, therefore a sample size of 32 was required per intervention.

### Test solution preparation

Trehalose solution spray (10%) was prepared, sterilized and packaged in the unlabeled 15-ml spray bottles. Briefly, trehalose powder (TREHA®, Nagase America LLC., USA) was weighed and dissolved in sterilized drinking water to produce 10% trehalose solution (10 g in 100 mL). Potassium metabisulfite was added as a preservative to the final concentration of 0.05% [12]. CMC solution spray was purchased from Pharmacy Department at King Chulalongkorn Memorial Hospital, and then refilled in unlabeled 15-ml spray bottles identical to trehalose solution spray bottles.

### Data collection

The research design was a block-randomized controlled trial. After enrollment, 70 participants were randomly and blindly assigned to use either 10% trehalose intervention, or CMC oral solution control group. Before intervention (day 0), the participants were interviewed for the XeQoLs questionnaire and demographic data. Then, unstimulated saliva was collected by spitting into a 50-mL Eppendorf tube for 5 min and was calculated to unstimulated salivary flow rate (mL/min) (AP). Salivary pH was measured with a pH meter (HI 98,100 Checker Plus, HANNA Instruments, Thailand). To prevent contamination and disease transmission, saliva volume was measured after centrifugation in the biosafety tissue culture hood (KP).

Previously, a random allocation sequence was generated using a random-numbers table with a block size of 10 based on an estimated number of participants per week. The bottles of oral spray were sequentially numbered (KP) to be given to participants. The bottle of oral spray had no label so that the allocation was concealed to both patients and the researcher (AP). Participants were then instructed to use oral spray 4 times a day, after each meal and before bedtime. Each time, two pumps (approximately 0.4 mL) were intraorally applied. After 14 days of intervention (day 14), all participants were interviewed by phone using the XeQoLs questionnaire (KP), and VAS scores were recorded as a primary outcome by one researcher (KP). Because of COVID-19 pandemic, only some participants came back for saliva collection post treatment. Salivary pH and unstimulated salivary flow rate were recorded as secondary outcomes (KP). The sequential numbers on the bottles remained blinded to the researcher (AP) throughout the data collection phase.

### Questionnaires

The study used the quality-of-life questionnaire for the primary outcome. The questionnaire was modified from Xerostomia-related Quality of Life scale (XeQoLs) [27–30] and translated into Thai language by 4 dentists [27]. The questionnaire consisted of 14 questions and divided into 4 dimensions: (1) 4 questions of physical, (2) 4 questions of pain/discomfort, (3) 3 questions of psychological, and (4) 3 questions of social dimension. The index of item objective congruence (IOC) was adjusted to ≥ 0.5 in every question and was tested in the same subjects (18 patients) for reliability measurement with Cronbach’s alpha of 0.81. The subjects were asked to reply in Visual Analogue Scale (VAS) for the perception of dryness. The VAS was scaled from 0 to 10, in which “0” was the most positive response and “10” was the most negative response for example, “0” for not dry at all and “10” for the worst imaginable dryness.

### Data analysis

As for the RCT, all statistical analyses were performed using SPSS software v25.0 (SPSS Inc. New York, NY, USA). Description of the subjects was carried out by descriptive statistics. Shapiro-Wilk test was used as a normality test. Wilcoxon Signed Ranks was used to compare the quality-of-life scores between before and after intervention in CMC and trehalose group. Mann-Whitney U test was used to compare the quality-of-life before-after difference scores between both treatment group. Chi-square test was used to compare the percentage of cases with concomitant chemotherapy between both treatment group. Independent T-test was used to compare the means of duration (month) post treatment and the means of parotid gland mean doses between ipsilateral and contralateral sides in each intervention group and between trehalose and CMC solution spray groups, unstimulated salivary flow rate and salivary pH between trehalose and CMC solution spray groups. Paired T-test was used to compare unstimulated salivary flow rate and salivary pH pre- and post-intervention in each treatment group. The significance level was set at 5%.

## Results

### Characteristics of the study population

Seventy patients previously diagnosed with HNC were enrolled after completing radiotherapy. Demographic data and clinical characteristics of study participants are shown in Table 1. CMC-based (control; n = 35) and trehalose-based oral sprays (intervention; n = 35) were randomly assigned to participants as described in materials and methods. The mean age of participants was 54.14 ± 13.89 years (range: 36–75) and 58.29 ± 14.75 years (range: 22–85) in CMC and trehalose intervention groups, respectively. Participants were 65.71% male and 34.29% female in CMC group, and 62.86% male and 37.14% female in trehalose group. Nasopharynx was the most frequent primary site in this study, 71.42% in CMC and 31.43% in trehalose group. While, the second common site was the oral cavity, 20% in CMC and 34.29% in trehalose group. Other primary cancer sites, including salivary gland, nasal cavity and paranasal sinus and larynx, were approximately 3% and 10% in CMC and trehalose group, respectively. Most participants were diagnosed with cancer progression, 48.57% in CMC and 37.14% in trehalose group were at stage III, and 40% of both groups were at stage IV. Most patients were treated using Intensity Modulated Radiation Therapy/Volumetric Modulated Radiation Therapy (IMRT/VMAT), while 5.71% and 8.57% received three-dimensional conformal radiation therapy (3D CRT). Approximately 91% and 66% in CMC and trehalose groups (*p* < 0.05), respectively, concomitantly received chemotherapy. The mean of time post-radiation was 7.06 ± 3.86 months, and 4.71 ± 3.41 months, for CMC and trehalose group (*p* < 0.05), respectively.

**Table 1:** Patient characteristics

Participant characteristics	Intervention	
	CMC (n = 35)	Trehalose (n = 35)
Age		
mean (years) ± SD	54.14 ± 13.89	58.29 ± 14.75
Range	36–75	22–85
Gender, n (%)		
Male	23 (65.71%)	22 (62.86%)
Female	12 (34.29%)	13 (37.14%)
Primary cancer site, n (%)		
Nasopharynx	25 (71.42%)	11 (31.43%)
Oral cavity	7 (20%)	12 (34.29%)
Salivary gland	1 (2.86%)	3 (8.57%)
Nasal cavity and paranasal sinus	1 (2.86%)	4 (11.43%)
Larynx	1 (2.86%)	4 (11.43%)
Thyroid	0 (0%)	1 (2.86%)
Stage of cancer, n (%)		
Stage I	1 (2.86%)	1 (2.86%)
Stage II	3 (8.57%)	7 (20%)
Stage III	17 (48.57%)	13 (37.14%)
Stage IV	14 (40%)	14 (40%)
Radiation technique, n (%)		
IMRT/VMAT^a^	33 (94.29%)	32 (91.43%)
3D CRT^b^	2 (5.71%)	3 (8.57%)
Duration after radiation (months)		
Mean ± SD	7.06 ± 3.86^c^	4.71 ± 3.41
Range	1–12	1–11
Concomitant chemotherapy, n (%)	32 (91.42%)^d^	23 (65.71%)

^a^ Intensity Modulated Radiation Therapy/Volumetric Modulated Radiation Therapy

^b^ Three-dimensional conformal radiation therapy

^c^ The mean duration after radiation of CMC was significantly higher than trehalose; Independent T test; *p* < 0.05

^d^ The percentage of patients receiving concomitant chemotherapy during CMC treatment was significantly higher than during trehalose treatment; Chi-square test; *p* < 0.05

To evaluate the irradiation effect on the SG, the parotid gland mean dose in the CMC and trehalose interventions was determined (Table 2). The mean dose on the ipsilateral side targeted for radiotherapy was significantly higher than the contralateral side (*p* < 0.001). Nonetheless, the parotid gland mean doses between CMC and trehalose groups were not statistically different (in either the ipsilateral or contralateral sides), suggesting a similar exposure to radiation in both groups.

**Table 2:** The absorbed radiation dose on parotid glands of both ipsilateral and contralateral sides in HNC patients receiving CMC-based or trehalose oral spray

Position related to HNC	Parotid gland mean dose (Gy) ± SD		*p*-value
	CMC	Trehalose	
Ipsilateral	28.59 ± 10.37	27.74 ± 15.67	0.791
Contralateral	23.70 ± 6.38	21.71 ± 12.17	0.396
*p*-value	0.000***	0.000***	

Independent T-test was used to compare the parotid gland mean doses between trehalose and CMC solution spray, and between ipsilateral and contralateral sides in each group. ***p < 0.001

### Trehalose oral spray increased salivary pH and unstimulated salivary flow rate

In pre-clinical trials perfomed ex vivo, 10% trehalose dose regimen supported the pro-acinar growth of the mouse fetal SG (Additional file 1**: Supplemental Fig. **1). Twenty participants were not able to come for the collection on day 14 (post-intervention) due to COVID-19 infection, hence the number of saliva samples was 25 for each intervention group. Table 3 displays the salivary pH and unstimulated salivary flow rate. At baseline (day 0), the mean salivary pH was 6.78 ± 0.64 in CMC and 6.91 ± 0.67 in trehalose group, respectively. At the same time point, unstimulated salivary flow rate was 0.17 ± 0.16 mL/min in CMC, and 0.16 ± 0.22 mL/min in trehalose group. The results on day 14 demonstrated that salivary pH and unstimulated salivary flow rate were increased after the interventions. In CMC group, salivary pH (*p* = 0.202) and unstimulated salivary flow rate (*p* = 0.146) were increased but not significantly. However, salivary pH and unstimulated salivary flow rate remarkably increased to 7.16 ± 0.56 (*p* = 0.033), and 0.20 ± 0.24 mL/min (*p* = 0.009), respectively, after using trehalose oral spray.

**Table 3:** Salivary pH and unstimulated salivary flow rate before and after treatment with CMC and trehalose solution spray

Intervention	Measurement (mean ± SD)	Day of data collection		*p*-value
		Day 0 (Before)	Day 14 (After)	
CMC	Salivary pH	6.78 ± 0.64	6.96 ± 0.7	0.20
	Unstimulated salivary flow rate (mL/min)	0.17 ± 0.16	0.19 ± 0.20	0.146
Trehalose	Salivary pH	6.91 ± 0.67	7.16 ± 0.56	0.033*
	Unstimulated salivary flow rate (mL/min)	0.16 ± 0.22	0.20 ± 0.24	0.009**

Paired T-test was used to compare unstimulated salivary flow rate and salivary pH pre- and post-intervention in each treatment group. *p < 0.05; **p < 0.01

### Radiation-induced xerostomia and quality of life is improved with both oral sprays

The quality-of-life scores from participants in the trehalose group were similar to the CMC group (Table 4). The responses from Q1 to Q4 regarding chewing (*p* = 0.015), swallowing (*p* = 0.000), talking (*p* = 0.002) and taste alteration (*p* = 0.000) were significantly improved with trehalose oral spray. Scores pertaining to pain and discomfort dimensions were significantly increased in Q5 (*p* = 0.000), Q6 (*p* = 0.003) and Q7 (*p* = 0.000). Psychological effects of oral dryness in Q9 (*p* = 0.005), Q10 (*p* = 0.004), and Q11 (*p* = 0.046) were significantly improved. However, the responses related to social interaction, Q12, Q13, Q14, did not change in participants in both the CMC and trehalose interventions.

**Table 4:** The median XeQoLs scores taken before and after treatment with CMC or trehalose solution spray

Questionnaire	XeQoLs score (Median ± IQR)					
	CMC group (n = 35)			Trehalose group (n = 35)		
	Before	After	*ρ*-value	Before	After	*ρ*-value
Part 1: Physical						
Q1: Rate your difficulty in chewing due to dryness	4.00 ± 7.00	3.00 ± 5.00	0.002**	0.00 ± 5.00	0.00 ± 4.00	0.015*
Q2: Rate your difficulty in swallowing food due to dryness	5.00 ± 4.00	3.00 ± 4.00	0.000***	5.00 ± 5.00	4.00 ± 4.00	0.000***
Q3: Rate your difficulty in talking due to dryness	3.00 ± 5.00	2.00 ± 4.00	0.003**	4.00 ± 5.00	3.00 ± 4.00	0.002**
Q4: Rate your taste alteration	5.00 ± 4.00	5.00 ± 4.00	0.000***	6.00 ± 4.00	5.00 ± 3.00	0.000***
Part 2: Pain / Discomfort						
Q5: Rate your feeling dry mouth	6.00 ± 3.00	4.00 ± 3.00	0.000***	6.00 ± 3.00	4.00 ± 2.00	0.000***
Q6: Rate the frequency of sipping water (nocturnal)	4.00 ± 5.00	3.00 ± 5.00	0.047*	4.00 ± 5.00	3.00 ± 3.00	0.003**
Q7: Rate the frequency of sipping water (daytime)	8.00 ± 2.00	6.00 ± 4.00	0.001**	0.00 ± 1.00	0.00 ± 1.00	0.000***
Q8: Rate your pain and discomfort	0.00 ± 2.00	0.00 ± 2.00	0.768	0.00 ± 1.00	0.00 ± 1.00	0.066
Part 3: Psychological						
Q9: My mouth/throat dryness interferes with my daily activity	2.00 ± 5.00	0.00 ± 4.00	0.011*	0.00 ± 5.00	0.00 ± 4.00	0.005**
Q10: My mouth/throat dryness makes me nervous	2.00 ± 5.00	0.00 ± 5.00	0.006**	0.00 ± 3.00	0.00 ± 3.00	0.004**
Q11: My mouth/throat dryness reduces my general happiness	0.00 ± 5.00	0.00 ± 4.00	0.019*	0.00 ± 0.00	0.00 ± 4.00	0.046*
Part 4: Social						
Q12: My mouth/throat dryness makes me uncomfortable speaking in front of other people	0.00 ± 2.00	0.00 ± 2.00	0.103	0.00 ± 0.00	0.00 ± 0.00	0.180
Q13: My mouth/throat dryness makes me uncomfortable when eating in front of other people	0.00 ± 4.00	0.00 ± 4.00	0.169	0.00 ± 0.00	0.00 ± 0.00	0.157
Q14: My mouth/throat dryness makes me from socializing (going out)	0.00 ± 2.00	0.00 ± 2.00	0.211	0.00 ± 0.00	0.00 ± 0.00	0.109

Wilcoxon Signed Ranks was used to compare the XeQoLs scores between before and after intervention in each treatment group. *p < 0.05; **p < 0.01, ***p < 0.001

XeQoLs scores revealed that the quality of life improved after using both oral sprays (Table 4). In the CMC intervention, the scores pertaining to the physical dimension, Q1-Q4, specifically chewing (*p* = 0.002), swallowing (*p* = 0.000), talking (*p* = 0.003) and taste alteration (*p* = 0.000) were significantly improved. Scores in pain and discomfort dimensions were also significantly better in questions Q5 (*p* = 0.000), Q6 (*p* = 0.047) and Q7 (*p* = 0.001). Psychological effects of oral dryness with negative impact on daily activities and nervousness was probed by Q9-Q11, and participant responses showed a significant improvement with both oral sprays (*p* < 0.05).

The XeQoLs before-after treatment delta scores, differences between pre- and post-treament, of the CMC and trehalose group were analyzed and reported (Table 5). The XeQoLs scores of all four dimensions were improved after intervention, and the differences were compared between CMC and trehalose groups. No item demonstrated statistical differences of the XeQoLs before-after treatment delta scores (*p* > 0.05).

**Table 5:** **The median of the XeQoLs before-after treatment difference scores taken from CMC- or trehalose-based oral spray treatment groups**

Questionnaire	Difference of XeQoL score (Before-After) Median ± IQR		
	CMC (n = 35)	Trehalose (n = 35)	*ρ*-value
Part 1: Physical			
Q1: Rate your difficulty in chewing due to dryness	0.00 ± 0.10	0.00 ± 0.00	0.320
Q2: Rate your difficulty in swallowing food due to dryness	0.00 ± 2.00	1.00 ± 2.00	0.381
Q3: Rate your difficulty in talking due to dryness	0.00 ± 1.00	0.00 ± 1.00	0.744
Q4: Rate your taste alteration	0.00 ± 2.00	0.00 ± 1.00	0.628
Part 2: Pain / Discomfort			
Q5: Rate your feeling dry mouth	2.00 ± 2.00	2.00 ± 2.00	0.841
Q6: Rate the frequency of sipping water (nocturnal)	0.00 ± 0.00	0.00 ± 1.00	0.421
Q7: Rate the frequency of sipping water (daytime)	0.00 ± 2.00	0.00 ± 2.00	0.781
Q8: Rate your pain and discomfort	0.00 ± 0.00	0.00 ± 0.00	0.685
Part 3: Psychological			
Q9: My mouth/throat dryness interferes with my daily activity	0.00 ± 0.00	0.00 ± 1.00	0.749
Q10: My mouth/throat dryness makes me nervous	0.00 ± 0.00	0.00 ± 1.00	0.753
Q11: My mouth/throat dryness reduces my general happiness	0.00 ± 0.00	0.00 ± 0.00	0.451
Part 4: Social			
Q12: My mouth/throat dryness makes me uncomfortable speaking in front of other people	0.00 ± 0.00	0.00 ± 0.00	0.655
Q13: My mouth/throat dryness makes me uncomfortable when eating in front of other people	0.00 ± 0.00	0.00 ± 0.00	0.977
Q14: My mouth/throat dryness makes me from socializing (going out)	0.00 ± 0.00	0.00 ± 0.00	0.645

Mann-Whitney U test was used to compare the XeQoLs before-after treatment difference scores between both treatment group

## Discussion

Up to this date, this is the first RCT showing that trehalose oral spray can improve dry mouth symptoms and xerostomia-related quality of life in a comparable fashion to a CMC spray. Saliva substitutes containing CMC are prescribed to relieve xerostomia and improve quality of life [27, 30]. Major therapeutical advantages of CMC are to coat and moisten the oral mucosa to improve dry mouth [31]. Alternatively, trehalose has been introduced as a moistening reagent in several health products [23, 32]. Trehalose was reported to maintain moisture, prevent atrophy of the tongue mucosa, and relieve the oral dryness discomfort after dental treatment in preliminary studies [17]. Nonetheless and up to now, the evidence supporting trehalose-based interventions to relieve dry mouth remains limited and comparison trials with CMC are lacking. This RCT compared the effects of trehalose and CMC oral sprays in patients with xerostomia. In radiation-induced xerostomia subjects, 10% trehalose oral spray significantly increased salivary pH and saliva volume after 14 days of use. XeQoLs scores revealed subjects’ positive responses and satisfaction with both trehalose and CMC oral sprays. Hence, our findings suggest that trehalose can be used as oral spray to effectively relieve dry mouth.

Though, this study has its own limitations. Subjects did receive different radiotherapy treatment regimens, however, the radiation exposure of the parotid gland (on both sides) to such regimens was similar among all subjects. The radiation exposure was not assessed in the submandibular and sublingual glands. Secondly, polypharmacy may have affected xerostomia levels since a comprehensive medication list could not be collected during this RCT. Thirdly, subjects may have been affected by COVID-19 pandemic and in such cases, they refused to commute to the hospital. To overcome this, questionnaires were performed over the phone, and this may introduce some information bias.

Radiation exposure after radiotherapy was evenly distributed between two intervention groups according to parotid gland radiation dose assessments. Mean parotid gland doses were 24–28 Gy in our study participants. Previous studies [33, 34] showed that radiation doses of 10–15 Gy start to induce a slight reduction of SG function, but such function can be gradually improved when the average radiation remains lower than 20–40 Gy. However, a strong effect can occur when the average radiation dose goes over 40 Gy [35, 36]. Therefore, mean parotid gland doses reported herein were consistent with SG hypofunction reflecting a decrease in salivary volume and pH. After 2 weeks of treatment, trehalose-based oral spray improved the salivary volume and pH. Our earlier ex vivo SG models support these observations, upon mimicking a topical administration route with 10–15% trehalose, the pro-acinar epithelial growth was maintained, and epithelial mitosis increased particularly with 10% trehalose (Additional file 1). These outcomes align with those from the in vitro study by Mori et al. [17]. Moreover, this latter study indicated that plaque pH never reached a critical pH after trehalose mouthwash was provided [25], suggesting that trehalose supports the neutralizing function of the saliva to balance dental plaque pH. Therefore, trehalose may not only moisten mucosal surfaces, but also have a biological effect on the function of SG relating to oral health.

Both CMC and trehalose oral sprays improved xerostomia-related quality of life particularly in physical, pain/discomfort, and psychological dimensions. However, major changes in the social dimension were not seen, which could relate to COVID-19 lockdown policies. The number of social events were dramatically reduced during the COVID-19 lockdown, and anxiety levels increased. Some participants avoided or were denied hospital visits for saliva collection, but the post intervention questionnaires were implemented through the phone.

The recovery of salivary gland function after radiation is related to radiation dose, radiation technique, concomitant chemotherapy and the involvement of radiation damage to salivary glands. Oral dryness is common, but improves over time in 10–30% of HNC patients [36]. Salivary flow rate and pH steeply decreased during the first month, then gradually increased within 3–6 months after radiotherapy [9]. Salivary gland function may recover after 12 months following completion of radiotherapy only, or concomitant chemotherapy treatment [36]. Then, unstimulated salivary flow rates slowly regain within 2 years although stimulated saliva levels remain low [37]. In this study, patients who completed radiotherapy for less than 1 month was excluded, and all data collection was performed within 12-month post radiation. Nonetheless, the CMC group had mean duration time after radiation and number of cases with concomitant chemotherapy higher than the trehalose group. The longer duration after radiation may reflect to lower effect on salivary gland function while the higher number of ongoing chemotherapy cases suggested higher effect on salivary gland function [38]. Despite the data being collected from different post-treatment periods, the mean pH and salivary flow rate were similar among the CMC and trehalose group. The pH and salivary flow rate was significantly improved only after using trehalose oral spray. Consequently, participants reported better effect of trehalose oral spray when the four dimensions were measured by XeQoLs scoring. Hence, while salivary gland functionally recover, the application of oral moisturizing agents including trehalose or CMC oral sprays are critical to improve oral health and quality of life as well as relieve other associated complications from radiotherapy and chemotherapy like oral mucositis.

Adverse effects of CMC when taken as a saliva substitute therapy have been reported [39]. An interaction between calcium and phosphate ions resulted in the formation of a chemical complex that decreases the demineralizing capabilities of saliva [39] and interferes with the dentin remineralization process [40, 41]. Remineralization effects by CMC interventions have been observed with higher salivary pH which improves when pH increases from 5.5 to 6.5 [40]. In addition, trehalose oral applications are more advantageous than CMC when it comes to subject’s overall satisfaction, taste perception and cost/affordability. Individuals using trehalose spray responded with higher satisfaction compared to those with CMC spray, due to the sweeter taste and less viscosity of trehalose [42, 43]. Nonetheless, in this RCT, a 10% trehalose oral spray improved xerostomia and the quality of life alike the CMC spray.

## Conclusion

Intraoral administration of 10% trehalose spray improved unstimulated salivary flow rate and pH in xerostomia subjects after radiotherapy for HNC. Equivalent with conventional CMC-based interventions, a trehalose oral spray can effectively relieve dry mouth symptoms in post-radiotherapy xerostomia patients with superior patients’ satisfaction. Thus, trehalose solution spray can be indicated as an alternative therapeutical option to relieve oral dryness and improve the quality of life in HNC patients post radiotherapy.

## Electronic supplementary material

Below is the link to the electronic supplementary material.

**Additional File 1**: **Supplement Figure 1** : Topical effects of trehalose in the mouse SG epithelia. Supplement Table 1: The mean XeQoLs scores taken before and after treatment with CMC or trehalose solution spray. **Supplement Table 2**: The mean difference in the XeQoLs scores taken before and after CMC or trehalose oral spray treatment

## Data Availability

The datasets generated and/or analysed during the current study are not publicly available because some data contained information that compromised the privacy of research participants, but are available from the corresponding author on reasonable request.

## References

[1] De Felice F, Polimeni A, Valentini V (2018). Radiotherapy controversies and prospective in head and neck cancer: a literature-based critical review. Neoplasia.

[2] Lastrucci L, Bertocci S, Bini V (2018). Xerostomia Quality of Life Scale (XeQoLS) questionnaire: validation of italian version in head and neck cancer patients. Radiol Med.

[3] Dirix P, Nuyts S, Van den Bogaert W (2006). Radiation-induced xerostomia in patients with head and neck cancer: a literature review. Cancer.

[4] Shiboski CH, Hodgson TA, Ship JA, Schiødt M. (2007) Management of salivary hypofunction during and after radiotherapy. Oral Surg Oral Med Oral Pathol Oral Radiol Endod 103 Suppl: S66.e1-19. doi: 10.1016/j.tripleo.2006.11.013. 10.1016/j.tripleo.2006.11.013 17379158

[5] Porter SR, Scully C, Hegarty AM (2004). An update of the etiology and management of xerostomia. Oral Surg Oral Med Oral Pathol Oral Radiol Endod.

[6] Guchelaar HJ, Vermes A, Meerwaldt JH (1997). Radiation-induced xerostomia: pathophysiology, clinical course and supportive treatment. Support Care Cancer.

[7] Deasy JO, Moiseenko V, Marks L, Chao KS, Nam J, Eisbruch A (2010). Radiotherapy dose-volume effects on salivary gland function. Int J Radiat Oncol Biol Phys.

[8] Lin CY, Ju SS, Chia JS, Chang CH, Chang CW, Chen MH (2015). Effects of radiotherapy on salivary gland function in patients with head and neck cancers. J Dent Sci.

[9] Dodds M, Roland S, Edgar M, Thornhill M (2015). Saliva. A review of its role in maintaining oral health and preventing dental disease. BDJ Team.

[10] Plemons JM, Al-Hashimi I, Marek CL (2014). Managing xerostomia and salivary gland hypofunction: executive summary of a report from the american Dental Association Council on Scientific Affairs. J Am Dent Assoc.

[11] Silvestre-Donat FJ, Miralles-Jorda L, Martınez-Mihi V (2004). Protocol for the clinical management of dry mouth. Med Oral.

[12] Ota Y, Morito A, Fujisawa K (2012). Evaluation of a moisturising micro-gel spray for prevention of cell dryness in oral mucosal cells: an in vitro study and evaluation in a clinical setting. Eur J Cancer Care.

[13] Davies AN (1997). The management of xerostomia: a review. Eur J Cancer Care.

[14] Murakami M, Nishi Y, Fujishima K (2016). Impact of types of moisturizers and humidity on the residual weight and viscosity of liquid and gel oral moisturizers. J Prosthodont.

[15] Mardani H, Ghannadi A, Rashnavadi B, Kamali R (2017). The effect of ginger herbal spray on reducing xerostomia in patients with type II diabetes. Avicenna J Phytomed.

[16] Hahnel S, Behr M, Handel G, Burgers R (2009). Saliva substitutes for the treatment of radiation-induced xerostomia-a review. Support Care Cancer.

[17] Mori Y, Yano F, Shimohata N, Suzuki S, Chung UI, Takato T (2010). Trehalose inhibits oral dryness by protecting the cell membrane. Int J Oral Maxillofac Surg.

[18] Elbein AD, Pan YT, Pastuszak I, Carroll D (2003). New insights on trehalose: a multifunctional molecule. Glycobiology.

[19] Guo N, Puhlev I, Brown DR, Mansbridge J, Levine F (2000). Trehalose expression confers desiccation tolerance on human cells. Nat Biotechnol.

[20] Matsuo T (2001). Trehalose protects corneal epithelial cells from death by drying. Br J Ophthalmol.

[21] Tanaka M, Machida Y, Nukina N (2005). A novel therapeutic strategy for polyglutamine diseases by stabilizing aggregation-prone proteins with small molecules. J Mol Med (Berl).

[22] Mancini RJ, Lee J, Maynard HD (2012). Trehalose glycopolymers for stabilization of protein conjugates to environmental stressors. J Am Chem Soc.

[23] Luyckx J, Baudouin C (2011). Trehalose: an intriguing disaccharide with potential for medical application in ophthalmology. Clin Ophthalmol.

[24] Mateo Orobia AJ, Casas Pascual P, Cristóbal Bescós J (2017). Effects of 3% trehalose as an adjuvant treatment after LASIK. Clin Ophthalmol.

[25] Neta T, Takada K, Hirasawa M. (2000) Low-cariogenicity of trehalose as a substrate. J Dent 2000 28:571–576. doi: 10.1016/s0300-5712(00)00038-5. 10.1016/s0300-5712(00)00038-5 11082525

[26] Gómez-Moreno G, Cabrera‐Ayala M, Aguilar‐Salvatierra A (2014). Evaluation of the efficacy of a topical sialogogue spray containing malic acid 1% in elderly people with xerostomia: a double‐blind, randomized clinical trial. Gerodontology.

[27] Sulistiyani E, Brimson JM, Chansaenroj A (2021). Epigallocatechin-3-gallate protects pro-acinar epithelia against salivary gland radiation injury. Int J Mol Sci.

[28] Boonroung T, Narongdej T, Vadcharavivad S (2011). A comparative study of carboxymethylcellulose and enzyme-containing saliva substitute on quality of life in head and neck cancer patients with self-reported postradiation-xerostomia. Thai Pharm Health Sci J.

[29] Henson BS, Inglehart MR, Eisbruch A, Ship JA (2001). Preserved salivary output and xerostomia-related quality of life in head and neck cancer patients receiving parotid-sparing radiotherapy. Oral Oncol.

[30] Meirovitz A, Murdoch-Kinch CA, Schipper M, Pan C, Eisbruch A (2006). Grading xerostomia by physicians or by patients after intensity-modulated radiotherapy of head-and-neck cancer. Int J Radiat Oncol Biol Phys.

[31] Oh DJ, Lee JY, Kim YK, Kho HS (2008). Effects of carboxymethylcellulose (CMC)-based artificial saliva in patients with xerostomia. Int J Oral Maxillofac Surg.

[32] Doan S, Bremond-Gignac D, Chiambaretta F (2018). Comparison of the effect of a hyaluronate-trehalose solution to hyaluronate alone on ocular surface Disease Index in patients with moderate to severe dry eye disease. Curr Med Res Opin.

[33] Hey J, Setz J, Gerlach R (2011). Parotid gland-recovery after radiotherapy in the head and neck region–36 months follow-up of a prospective clinical study. Radiat Oncol.

[34] Blanco AI, Chao KS, El Naqa I (2005). Dose-volume modeling of salivary function in patients with head-and-neck cancer receiving radiotherapy. Int J Radiat Oncol Biol Phys.

[35] Chao KS, Deasy JO, Markman J (2001). A prospective study of salivary function sparing in patients with head-and-neck cancers receiving intensity-modulated or three-dimensional radiation therapy: initial results. Int J Radiat Oncol Biol Phys.

[36] Likhterov I, Ru M, Ganz C (2018). Objective and subjective hyposalivation after treatment for head and neck cancer: long-term outcomes. Laryngoscope.

[37] Jensen SB, Pedersen AML, Vissink A (2010). A systematic review of salivary gland hypofunction and xerostomia induced by cancer therapies: prevalence, severity and impact on quality of life. Support Care Cancer.

[38] Vistoso MA, Polonsky G, Shiboski C (2022). Salivary gland dysfunction secondary to Cancer Treatment. Front Oral Health.

[39] Meyer-Lueckel H, Tschoppe P, Kielbassa AM (2006). Linseed based saliva substitutes and their effect on mineral dissolution of predemineralized bovine dentin in vitro. J Dent.

[40] Tschoppe P, Meyer-Lueckel H, Kielbassa AM (2008). Effect of carboxymethylcellulose-based saliva substitutes on predemineralised dentin evaluated by microradiography. Arch Oral Biol.

[41] Meyer-Lückel H, Kielbassa AM (2006). Influence of calcium phosphates added to mucin-based saliva substitutes on bovine dentin. Quintessence Int.

[42] Portmann M-O, Birch G (1995). Sweet taste and solution properties of α,α-trehalose. J Sci Food Agri.

[43] Vissink A, Waterman HA, s-Gravenmade EJ, Panders AK, Vermey A (1984). Rheological properties of saliva substitutes containing mucin, carboxymethylcellulose or polyethylenoxide. J Oral Pathol.

