# Supplementary material for: Trehalose versus carboxymethylcellulose oral spray for relieving radiation-induced xerostomia in head and neck cancer patients: a randomized controlled trial
Source: BMC Oral Health. 2023 May 13;23:288. doi: 10.1186/s12903-023-02966-4 (PMC10182540; doi:10.1186/s12903-023-02966-4)
Supplement: Supplementary file 1 — Additional File 1: Supplement Figure 1 : Topical effects of trehalose in the mouse SG epithelia. Supplement Table 1: The mean XeQoLs scores taken before and after treatment with CMC or trehalose solution spray. Supplement Table 2: The mean difference in the XeQoLs scores taken before and after CMC or trehalose oral spray treatment [file 12903_2023_2966_MOESM1_ESM.docx]

**ADDITIONAL FILE 1**

**Supplement Figure 1 : Topical effects of trehalose in the mouse SG epithelia**

*Materials and methods to study salivary gland epithelial growth ex vivo*

All laboratory experimental procedures were approved by the Institutional Biosafety Committee at the Chulalongkorn University Faculty of Dentistry (Certificate number: DENT CU-IBC 006/2019) and animal protocol was approved by Institutional Animal Care and Use Committee (Certificate number: 1973004). All reagents were purchased from Sigma and Thermo Fisher Scientific unless otherwise noted.

The *ex vivo* submandibular glands (SG) derived from post-mortem embryos of ICR mice at embryonic day 14-15 [31] was cultured in growth media (GM) prepared in DMEM/F12 media without phenol red with 1 mM CaCl2, 100 U/mL penicillin, 100 μg/mL streptomycin, 150 μg/mL ascorbic acid, and 100 μg/mL human holo-transferrin. To mimic topical drug administration, a polycarbonate track-etched hydrophilic 0.1 µm porous membrane (Whatman™, Cytiva, Marlborough, MA, USA) was coated with GM supplemented with a range of trehalose concentrations (5-20%). SG were placed at the center of the coated membrane before GM was added underneath and incubated at 37 ^o^C under 5% CO_2_. Media was changed 50% every day and glands were cultured for a total of 48h. SG epithelial growth was determined at baseline, 24h and 48h by using the Spooner's ratio formula. This ratio was calculated by determining the epithelial bud counts from each time point divided by initial bud counts. For whole-mount immunofluorescence staining, SG were fixed at 48 h of culture in 4% paraformaldehyde, then incubated with 0.1% Triton X in PBS for immunofluorescence as previously reported [31]. Briefly, glands were incubated overnight with primary antibody against Ki67 (dilution 1:200, Invitrogen, PA5-19462), followed by Alexa Fluor 488 secondary antibody at room temperature. After such, Hoechst 33342 was used as a nuclear stain.

Regarding *ex vivo* experiments, data was analyzed using one-way ANOVA with Dunnett's post-hoc tests at each time point, which were run using GraphPad Prism version 6 (San Diego, CA, USA), where significance level was set at 5%. Error bars in plotted graph correspond to standard deviations.

*Results*

The *ex vivo* SG model was used to confirm the efficacy of 10% trehalose before clinical trial. The results demonstrated that 10-15% trehalose-coated membranes supported SG pro-acinar epithelial growth similar to control glands on membranes without trehalose as there were no statistical significant differences (Supplement Figure 1A), but such outcomes are more remarkable for 10% trehalose. These findings are confirmed by the bright field micrographs where one can depict an increased number of pro-acinar epithelial buds and a larger SMG gland size with 10% trehalose treatment (Supplement Figure 1B). Fluorescence imaging after Ki67 immunofluorescence (Figure 1B) also confirmed an increase of this proliferation marker in the pro-acinar buds of SG treated with 10% as well as 15% trehalose.


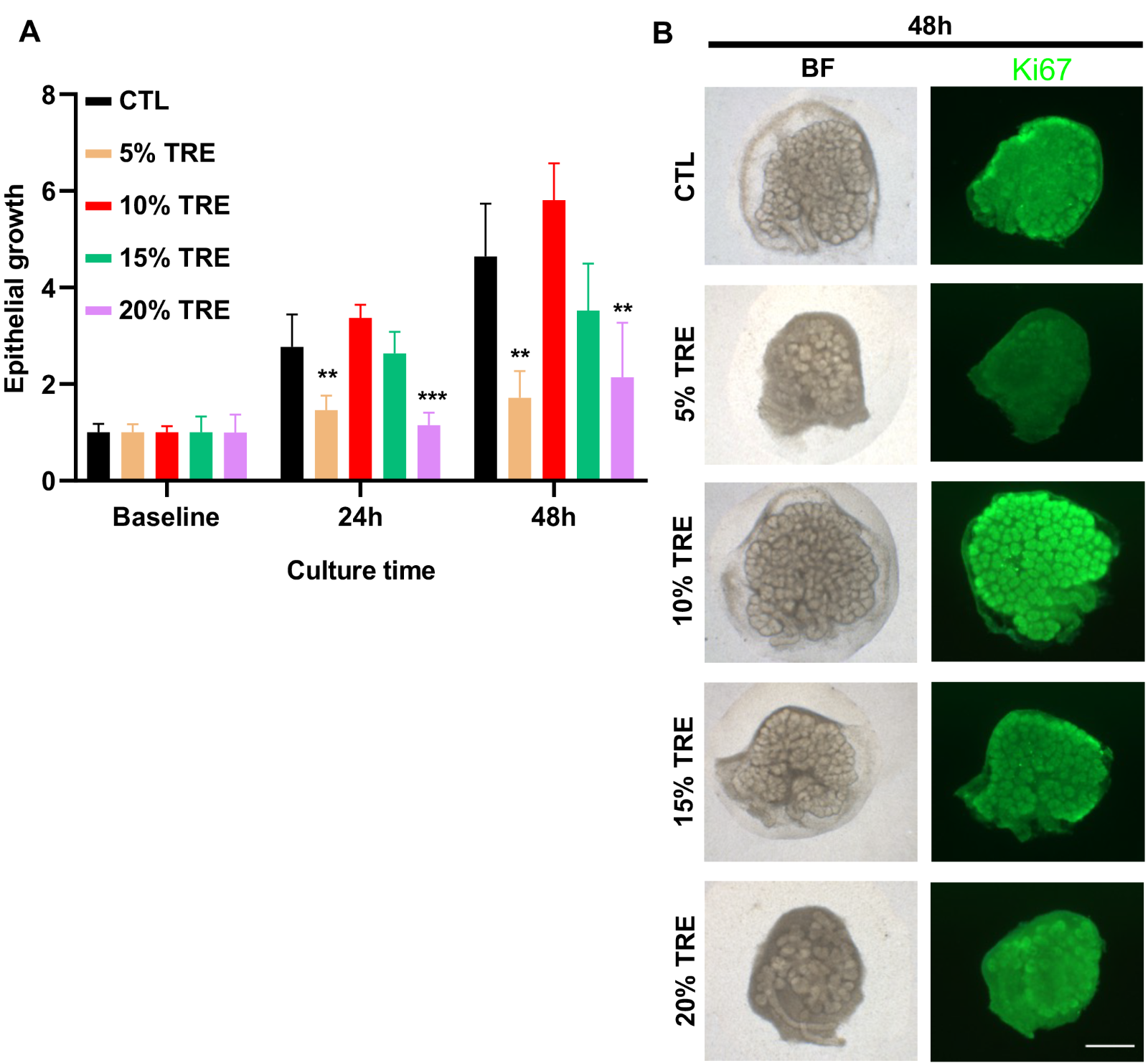

Supplement Figure 1. Topical effects of trehalose support the SMG epithelial growth. Trehalose (10-15%) coated on cultured surface supported SMG epithelial growth at 24 and 48 h. (A) SMG branching morphogenesis index or epithelial growth was calculated as described in Materials and Methods. Statistical analysis was performed by one-way ANOVA, post hoc: Dunnett, N=4; **p < 0.005, ***p < 0.0005. Each data point represents the mean ± SD. (B) A representative of the bright field imaging of SMG cultured on trehalose-coated membrane. Mag. 4x, Scale bar: 400 µm, and the immunofluorescence staining for a marker of proliferation (Ki67), Mag. 4x and 20x, Scale bar: 100 µm

**Supplement Table 1.**  **The mean XeQoLs scores taken before and after treatment with CMC or trehalose solution spray**

| Questionnaire | XeQoLs score (Mean±SD) | | | | | |
| --- | --- | --- | --- | --- | --- | --- |
|  | CMC group (n=35) | | | Trehalose group (n=35) | | |
|  | Before | After | *ρ*-value | Before | After | *ρ*-value |
| Part 1: Physical |  |  |  |  |  |  |
| Q1: Rate your difficulty in chewing due to dryness | 3.69±3.04 | 3.17±2.70 | 0.002** | 2.43±2.92 | 2.14±2.71 | 0.015* |
| Q2: Rate your difficulty in swallowing food due to dryness | 4.57±2.59 | 3.69±2.42 | 0.000*** | 4.86±3.02 | 3.77±2.59 | 0.000*** |
| Q3: Rate your difficulty in talking due to dryness | 3.00±2.68 | 2.63±2.48 | 0.003** | 3.11±2.54 | 2.74±2.28 | 0.002** |
| Q4: Rate your taste alteration | 5.29±2.82 | 4.40±2.64 | 0.000*** | 5.89±2.52 | 5.20±2.48 | 0.000*** |
| Part 2: Pain / Discomfort |  |  |  |  |  |  |
| Q5: Rate your feeling dry mouth | 5.94±2.01 | 3.86±2.02 | 0.000*** | 6.23±1.85 | 4.11±1.69 | 0.000*** |
| Q6: Rate the frequency of sipping water (nocturnal) | 3.29±3.17 | 2.97±2.91 | 0.047* | 4.29±2.59 | 3.66±2.29 | 0.003** |
| Q7: Rate the frequency of sipping water (daytime) | 6.66±2.80 | 5.77±2.89 | 0.001** | 6.66±1.94 | 5.71±2.14 | 0.000*** |
| Q8: Rate your pain and discomfort | 1.06±2.00 | 1.09±2.13 | 0.768 | 1.20±2.32 | 1.00±2.00 | 0.066 |
| Part 3: Psychological |  |  |  |  |  |  |
| Q9: My mouth/throat dryness interferes with my daily activity | 2.51±2.71 | 2.11±2.59 | 0.011* | 1.97±2.82 | 1.63±2.41 | 0.005** |
| Q10: My mouth/throat dryness makes me nervous | 2.49±2.73 | 2.09±2.61 | 0.006** | 1.80±2.56 | 1.43±2.24 | 0.004** |
| Q11: My mouth/throat dryness reduces my general happiness | 2.03±2.96 | 1.80±2.74 | 0.019* | 1.06±2.31 | 0.94±2.16 | 0.046* |
| Part 4: Social |  |  |  |  |  |  |
| Q12: My mouth/throat dryness makes me uncomfortable speaking in front of other people | 1.43±2.38 | 1.31±2.23 | 0.103 | 0.91±2.38 | 0.83±2.11 | 0.180 |
| Q13: My mouth/throat dryness makes me uncomfortable when eating in front of other people | 2.03±3.20 | 1.89±3.08 | 0.169 | 1.00±2.36 | 0.89±2.10 | 0.157 |
| Q14: My mouth/throat dryness makes me from socializing (going out) | 1.63±3.03 | 1.51±2.91 | 0.211 | 0.97±2.57 | 0.80±2.18 | 0.109 |

Wilcoxon Signed Ranks was used to compare the XeQoLs scores between before and after intervention in each treatment group. **p* < 0.05; ***p* < 0.01, ****p* < 0.001

**Supplement Table 2.**  **The mean difference in the XeQoLs scores taken before and after CMC or trehalose oral spray treatment**

| Question | Difference of XeQoL score (Before-After) Mean ± SD | | |
| --- | --- | --- | --- |
|  | CMC  (n = 35) | Trehalose  (n = 35) | *ρ*-value |
| Part 1: Physical |  |  |  |
| Q1: Rate your difficulty in chewing due to dryness | 0.51±0.89 | 0.28±0.62 | 0.320 |
| Q2: Rate your difficulty in swallowing food due to dryness | 0.89±1.16 | 1.09±1.07 | 0.381 |
| Q3: Rate your difficulty in talking due to dryness | 0.37±0.69 | 0.37±0.60 | 0.744 |
| Q4: Rate your taste alteration | 0.89±1.13 | 0.69±0.87 | 0.628 |
| Part 2: Pain / Discomfort |  |  |  |
| Q5: Rate your feeling dry mouth | 2.09±1.34 | 2.00±1.16 | 0.841 |
| Q6: Rate the frequency of sipping water (nocturnal) | 0.43±0.85 | 0.63±1.09 | 0.421 |
| Q7: Rate the frequency of sipping water (daytime) | 1.06±1.33 | 0.94±1.21 | 0.781 |
| Q8: Rate your pain and discomfort | 0.14±0.55 | 0.20±0.63 | 0.685 |
| Part 3: Psychological |  |  |  |
| Q9: My mouth/throat dryness interferes with my daily activity | 0.40±0.88 | 0.34±0.68 | 0.749 |
| Q10: My mouth/throat dryness makes me nervous | 0.40±0.81 | 0.37±0.65 | 0.753 |
| Q11: My mouth/throat dryness reduces my general happiness | 0.23±0.55 | 0.11±0.32 | 0.451 |
| Part 4: Social |  |  |  |
| Q12: My mouth/throat dryness makes me uncomfortable speaking in front of other people | 0.11±0.40 | 0.09±0.37 | 0.655 |
| Q13: My mouth/throat dryness makes me uncomfortable when eating in front of other people | 0.14±0.60 | 0.11±0.47 | 0.977 |
| Q14: My mouth/throat dryness makes me from socializing (going out) | 0.11±0.53 | 0.17±0.62 | 0.645 |

Mann-Whitney U test was used to compare the XeQoLs before-after treatment difference scores between both treatment group.
